# Legion: Best-First Concolic Testing (Competition Contribution)

**DOI:** 10.1007/978-3-030-45234-6_31

**Published:** 2020-03-13

**Authors:** Dongge Liu, Gidon Ernst, Toby Murray, Benjamin I. P. Rubinstein

**Affiliations:** 8 University of Paderborn, Paderborn, Germany; 9 ICREA, Open University of Catalonia, Barcelona, Spain; 10 University of Melbourne, Melbourne, Australia; 11 LMU Munich, Munich, Germany

**Keywords:** Symbolic Execution, Fuzzing, Monte Carlo Search

## Abstract

Legion is a grey-box coverage-based concolic tool that aims to balance the complementary nature of fuzzing and symbolic execution to achieve the best of both worlds. It proposes a variation of *Monte Carlo tree search (MCTS)* that formulates program exploration as sequential decision-making under uncertainty guided by the best-first search strategy. It relies on *approximate path-preserving fuzzing*, a novel instance of constrained random testing, which quickly generates many diverse inputs that likely target program parts of interest. In Test-Comp 2020 [1], the prototype performed within 90% of the best score in 9 of 22 categories.

## 1 Test-Generation Approach

Coverage testing aims to traverse all execution paths of the program under test to verify its correctness. Two traditional techniques for this task, *symbolic execution* [6] and *fuzzing* [7] are complementary in nature [5].

Consider exploring the program `Ackermann02` in Fig. 1 from the Test-Comp benchmarks as an example. Symbolic execution can compute inputs to penetrate the choke point (line 10) to reach the "rare branch" (lines 14/15), but then becomes unnecessarily expensive in solving the exponentially growing constraints from repeatedly unfolding the recursive function `ackermann`. By comparison, even though very few random fuzzer-generated inputs pass the choke point, the high speed of fuzzing means the "rare branch" will be quickly reached.

The following research question arises when exploring the program space in a conditional branch: Will it be more efficient to focus on the space under the constraint, or to flood both branches with unconstrained inputs, to target the internals of `log(m,n)` in line 11 at the same time?

LEGION[3] introduces *MCTS-guided program exploration* as a principled answer to this question, tailored to each program under test. For a program like

⋆ This research was supported by Data61 under the Defence Science and Technology Group's Next Generation Technologies Program.

⋆⋆ Jury Member

3 The name LEGION comes from the Marvel fictional character who changes personalities for different needs, to reflect the strategy adaption depending on the program.

H. Wehrheim and J. Cabot (Eds.): FASE 2020, LNCS 12076, pp. 545–549, 2020.
https://doi.org/10.1007/978-3-030-45234-6_31

```c
int ackermann(int m, int n) {
  if (m==0) return n+1;
  if (n==0) return ackermann(m-1,1);
  return ackermann(m-1,ackermann(m,n-1));
}

void main() {
  int m = input(), n = input();
  // choke point
  if (m < 0 || m > 3) || (n < 0 || n > 23) {
    log(n,m);              // common branch
    return;
  } else {
    int r = ackermann(m,n); // rare branch
    assert(m < 2 || r >= 4);
  }
}
```

Fig. 1: `Ackermann02.c`

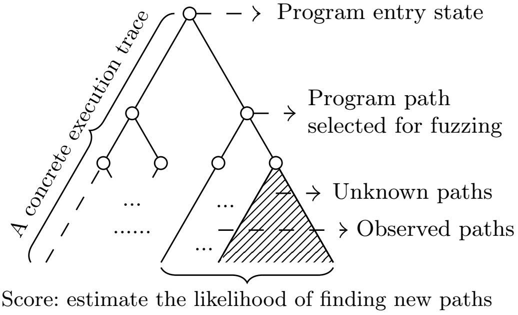

Fig. 2: MCTS-guided fuzzing in Legion

Fig. 2, Legion estimates the expectation of finding new paths by the UCT score (upper confidence bound for trees), a successful approach for games [3], aiming to balance *exploration* of program space (where success is still uncertain) against *exploitation* of partial results (that appear promising already). Code behind rare branches is targeted by *approximate path-preserving fuzzing* to efficiently generate diverse inputs for a specific sub-part of the program.

Legion's MCTS iteratively explores a tree-structured search space, whose nodes represent partial execution paths. On each iteration, Legion selects a *target* node by recursively descending from the root along the highest scoring child, stopping when a parent's score exceeds its childrens'. A node's score is based on the ratio of the number of distinct vs. all paths observed passing through it, but nodes selected less often in the past are more likely to be chosen. Then, approximate path-preserving fuzzing is applied to explore the target node. The resulting execution traces are recorded and integrated into the tree.

Approximate path-preserving fuzzing (APPF) quickly generates inputs that likely follow the target program path, and therefore is crucial for Legion's efficiency. Legion's APPF implementation extends the QuickSampler [4] technique, which is a recent mutation-based algorithm that expands a small set of constraint solutions to a larger suite of likely solutions. Legion extends QuickSampler from propositional logic to bitvector path constraints.

## 2 Tool Description & Configuration

We implemented Legion as a prototype in `Python3` on top of the symbolic execution engine `angr` [8]. We have extended its solver backend, `claripy`, by the approximate path-preserving fuzzing algorithm, relying on the optimizer component of Z3 [2]. Binaries are instrumented to record execution traces.

**Installation.** Download and unpack the competition archive (commit `b2fc8430`): `https://gitlab.com/sosy-lab/test-comp/archives-2020/blob/master/2020/legion.zip`

Legion requires `Python3` with `python-setuptools` installed, and `gcc-multilib` for the compilation of C sources. Necessary libraries compiled for Ubuntu 18.04

are included in the subfolder `lib` (modified versions of `angr`, `claripy` and their dependencies). The archive contains the main executable, `Legion.py`, and a wrapper script, `legion-sv` that includes `lib` into `PYTHONPATH`. The version tag is `0.1-testcomp2020`, options can be shown with `python3 ./Legion.py --help`.

**Configuration.** In the competition, we ran `./legion-sv` with these parameters:

| | |
|---|---|
| `--save-tests` | save test cases as `xml` files in Test-Comp format |
| `--persistent` | keep running when no more symbolic solutions are found (mitigates issue with dynamic memory allocations) |
| `--time-penalty 0` | do not penalise a node for expensive constraint-solving (experimental feature, not yet evaluated) |
| `--random-seed 0` | fix the random seed for deterministic result |
| `--symex-timeout 10` | limit symbolic execution and constraint solving to 10s |
| `--conex-timeout 10` | limit concrete binary execution to 10s |

In the category `cover-branches`, we additionally use this flag:

| | |
|---|---|
| `--coverage-only` | don't stop when finding an error |

Finally, `-32` and `-64` indicate whether to use 32 or 64 bits (this affects binary compilation and the sizes for nondeterministic values of types `int`, ...).

**Participation.** Legion participates in all categories of Test-Comp 2020.

**Software Project and Contributors.** Legion is principally developed by Dongge Liu, with technical and conceptual contributions by all authors of this paper. Legion will be made available at https://github.com/Alan32Liu/Legion.

## 3 Discussion

Legion is competitive in many categories of Test-Comp 2020, achieving within 90% of the best score in 2 of 9 error categories and 7 of 13 coverage categories.

```c
void main( ) {
  int N=100000, a1[N], a2[N], a3[N], i;
  for (i=0; i<N; i++)
    a1[i] = input(); a2[i] = input();
  for(i=0; i<N; i++) a3[i] = a1[i];
  for(i=0; i<N; i++) a3[i] = a2[i];
  for(i=0; i<N; i++) assert(a1[i] == a3[i]);
}
```

Fig. 3: `standard_copy2_ground-1.c`

Legion's instrumentation and exploration algorithm can accurately model the program. Consider the benchmark `standard_copy2_ground-1.c` in Fig. 3. With a single symbolic execution through the entire program over a trace found via initial random inputs, Legion understands that all guards of the `for` loops can only evaluate in one way, and so omits them from the selection phase. It does discover that the assertion inside the last loop contributes interesting decisions, however, and will come up with two different ways to evaluate the comparison `a1[i] == a3[i]`, one of which triggers the error. With such an accurate model in combination with its principled MCTS search strategy, Legion is particularly good at covering corner cases in deep loops: All other tools failed to score full marks in `standard_copy*_ground-*.c` benchmarks, but Legion succeeded in 9 out of 18. We can furthermore solve benchmarks where pure constraint solving fails, e.g., when the solver times out on hard constraints of complex paths we label the respective branches for pure random exploration.

While instrumentation provides accurate information on the program, its currently naive implementation significantly slows down the concrete execution of programs with long execution traces. We mitigate this weakness by setting a time limit on the concrete executions. As a consequence, inputs that correspond to long concrete execution are not saved. In the future, we plan to explore Intel's PIN tool, which offloads binary tracing into the CPU with negligible overhead.

Legion inherits some limitations from `angr` as a symbolic execution backend. Some benchmarks, such as `array-tiling/mbpr5.c`, dynamically allocate memory with a symbolic size that depends on the input. `angr` eagerly concretises this value, producing unsatisfiable path constraints for a feasible execution path. Legion detects this inconsistency as soon as it encounters the feasible path and omits the erroneous node from selection. This helps e.g. on `bubblesort-alloca-1.c` where Legion achieved full coverage (in contrast to most other participants) despite the dynamic allocations.

Legion performed poorly on benchmark sets `bitvector` and `ssh-simplified`. These programs have long sequences of equality constraint that are hard to satisfy with fuzzing. This happens to be an extreme example of the parent-child trade-off that Legion intends to balance where fuzzing the parent gives nearly no reward. This could potentially be mitigated by decreasing Legion's exploration ratio in the UCT score, but we have not attempted such fine-tuning.

Another problem is allocations when loop counters or array sizes are randomly chosen very large in 64 bit mode, leading to excessively long concrete execution traces that cause timeouts or memory exhaustion. We plan to periodically prune the in-memory representation of the tree in the future.
